# Promising Advances in LINC01116 Related to Cancer

**DOI:** 10.3389/fcell.2021.736927

**Published:** 2021-10-14

**Authors:** Yating Xu, Xiao Yu, Menggang Zhang, Qingyuan Zheng, Zongzong Sun, Yuting He, Wenzhi Guo

**Affiliations:** ^1^Department of Hepatobiliary and Pancreatic Surgery, The First Affiliated Hospital of Zhengzhou University, Zhengzhou, China; ^2^Key Laboratory of Hepatobiliary and Pancreatic Surgery and Digestive Organ Transplantation of Henan Province, The First Affiliated Hospital of Zhengzhou University, Zhengzhou, China; ^3^Open and Key Laboratory of Hepatobiliary and Pancreatic Surgery and Digestive Organ Transplantation at Henan Universities, Zhengzhou, China; ^4^Henan Key Laboratory of Digestive Organ Transplantation, Zhengzhou, China; ^5^Department of Obstetrics and Gynecology, The Third Affiliated Hospital of Zhengzhou University, Zhengzhou, China

**Keywords:** long non-coding RNA, LINC01116, human cancers, prognosis, chemoresistance

## Abstract

Long non-coding RNAs (lncRNAs) are RNAs with a length of no less than 200 nucleotides that are not translated into proteins. Accumulating evidence indicates that lncRNAs are pivotal regulators of biological processes in several diseases, particularly in several malignant tumors. Long intergenic non-protein coding RNA 1116 (LINC01116) is a lncRNA, whose aberrant expression is correlated with a variety of cancers, including lung cancer, gastric cancer, colorectal cancer, glioma, and osteosarcoma. LINC01116 plays a crucial role in facilitating cell proliferation, invasion, migration, and apoptosis. In addition, numerous studies have recently suggested that LINC01116 has emerged as a novel biomarker for prognosis and therapy in malignant tumors. Consequently, we summarize the clinical significance of LINC01116 associated with biological processes in various tumors and provide a hopeful orientation to guide clinical treatment of various cancers in future studies.

## Introduction

Cancer is one of the leading causes of death (Seow et al., 2020; Wang et al., 2020d; Buneviciene et al., 2021; Eloranta et al., 2021) worldwide and threatens human health and social happiness. Despite the advancements (Blessin et al., 2020) in clinical diagnosis and treatment of malignancies (Jung et al., 2020; Grady et al., 2021; Hussain et al., 2021), most patients still have a poor prognosis, and the overall survival (OS) rate remains low. Due to the lack of early diagnostic biomarkers, most cancers progress to the terminal stage. Therefore, it is urgent to understand the underlying molecular mechanisms in cancer, which is crucial for finding effective early diagnostic biomarkers and therapeutic methods.

Owing to the development of multiple RNA detection techniques (Shu et al., 2021; Toden et al., 2021; Wang et al., 2021; Zhong et al., 2021a), most RNAs have been found to lack the capacity to encode proteins. Although these RNAs do not directly translate into proteins (Gupta et al., 2020), driving evidence clarifies that they play an essential role in biological functions (Chen et al., 2020b; Jantrapirom et al., 2021; Jin et al., 2021; Lai et al., 2021; Zhang et al., 2021b). Such is the case of long non-coding RNAs (lncRNAs), which are no less than 200 nucleotides and participate in the initiation and progression of various diseases, especially cancers (Chen et al., 2020a; Huang et al., 2021; Kotani et al., 2021; Luo et al., 2021; Teng et al., 2021). Abnormal expression of these lncRNAs is involved in a variety of biological processes in tumors *via* regulation of gene expression that affects tumor size, metastasis, pathological stage, and prognosis in patients (Yin et al., 2018; Shuai et al., 2020; Li et al., 2021a; Zheng et al., 2021; Zhong et al., 2021b). Moreover, studies have confirmed that lncRNAs serve as competitive endogenous RNAs (ceRNAs) and could sponge microRNAs to regulate the expression of messenger RNA, which provides a promising direction for exploring the complicated molecular mechanisms of malignancies.

Long intergenic non-protein coding RNA 1116 (LINC01116), located in the 2q31.1 region, is currently reported to be an extraordinary regulator of proliferation, migration, and invasion of cancer cells (Meng et al., 2020; Cui et al., 2021; Lou et al., 2021; Ren et al., 2021; Zhang et al., 2021a). High expression of LINC01116 was identified in malignant tumors; LINC01116 might participate in tumorigenesis. For instance, previous studies have demonstrated that hepatocellular carcinoma (HCC) patients with LINC01116 overexpression generally have a dismal survival time (Jiang et al., 2019). Further studies verified that LINC01116 promoted oral squamous cell carcinoma (OSCC) proliferation, migration, and invasion (Chen et al., 2019). In contrast, knockdown of LINC01116 positively inhibited the proliferation of prostate cancer cells. Additionally, numerous reports have shown that LINC01116 functions as a major regulator of lung cancer (LC), gastric cancer (GC), colorectal cancer (CRC), glioma, osteosarcoma, glioma, head and neck squamous cell carcinoma (HNSC), epithelial ovarian cancer (EOC), and breast cancer (BC).

In this review, we highlight the latest studies concerning LINC01116, its abnormal expression related to clinical characteristics, and its influence on multiple biological functions of cancers. The present review could guide the further discovery of prospective and creative therapeutic targets.

## Expression and Clinical Characteristics of LINC01116 in Various Cancer Types

Numerous studies have elucidated the significance of LINC01116 in malignancy. Therefore, we review the specific process in Table 1, which shows how LINC01116 expression exerts its impact on a variety of cancers.

**TABLE 1:** Expression and effect on clinical characters of LINC01116 in different cancer types.

**Cancer type**	**Sample**	**Expression**	**Clinical characters**	**Prognosis**	**PMID**
Lung cancer	594 cases	High	Advanced tumor stage	Poor	32913506
	84 cases	High	Prognosis	Poor	32913506
	62 cases	High	Advanced tumor stage	Poor	33535997
	318 cases	High	Prognosis	Poor	33987935
Osteosarcoma	104 cases	High	Higher clinical stage	Poor	31486480
Glioma	135 cases	High	Early tumor metastasis	Poor	31933922
	37 cases	High	Prognosis	Poor	32358484
Gastric cancer	73 cases	High	Prognosis	Poor	32141549
	76 cases	High	Prognosis	Poor	31632064
Colorectal cancer	62 cases	High	Lower clinical stage	Poor	33116633
	80 cases	High	Prognosis	Poor	33499872
Oral squamous cell carcinoma	58 cases	High	Prognosis	Poor	31308744
Neck squamous cell carcinoma	44 cases	High	/	/	31452270
Prostate adenocarcinoma cell	15 cases	High	/	/	28131897
Epithelial ovarian cancer	90 cases	High	Prognosis	Poor	30178832
Breast cancer	94 cases	High	Advanced tumor stage	Poor	29687853

### Lung Cancer

Lung cancer is predominant worldwide and its incidence rate is the highest in men and the second in women (Bade and Dela Cruz, 2020; Chen et al., 2021; Ma et al., 2021; Yuan et al., 2021). Zeng et al. (2020) showed that LINC01116 was overexpressed in LC tumor tissues compared to normal adjacent tissues. LINC01116 expression is high in LC patients, and they generally have more unsatisfactory outcomes than the others. Thus, previous reports have suggested that LINC01116 is an independent prognostic factor in LC. Furthermore, the expression of LINC01116 was shown to be high in patients with advanced tumor stages. For instance, recent studies showed that a considerable number of patients with low expression of LINC01116 were generally diagnosed with TNM I rather than TNM I/III. These results indicate that LINC01116 can be considered as a latent regulator that participates in the progression of metastasis and invasiveness in LC (Shang et al., 2021). Additionally, silencing of LINC01116 reverses this effect. Eventually, LINC01116 plays crucial roles in tumor processes and could provide an orientation for being diagnostic and prognostic markers of LC, but its actual situation of clinical application still requires massive clinical and basic research.

### Gastric Cancer

Gastric cancer is the 4th most prevalent malignant tumor. Due to the lack of early diagnostic markers, patients are commonly diagnosed at terminal stages, with tumors that have metastasized to proximal or even remote regions in the body (Lin et al., 2019; Arnold et al., 2020; Ascherman et al., 2021; Puliga et al., 2021). Su et al. (2019) discovered that LINC01116 and CASC11 were upregulated in GC tissues, compared with cancer-adjacent tissues, and were positively correlated with clinical stages. In addition, the overexpression of LINC01116 and CASC11 was found to collectively increase the migration and invasion of GC cells. In contrast, low expression of LINC01116 was found to be associated with suppressed metastasis and invasiveness in GC patients. Moreover, CASC11 silencing alleviated the overexpression of LINC01116. Additionally, Chen et al. (2020c) demonstrated that patients with abundant expression of LINC01116 in GC cells generally have shorter survival time than those with low expression levels, and that the inhibition of LINC01116 expression could impede the proliferation of GC cells. These findings provide a novel direction for the diagnosis and treatment of GC.

### Colorectal Cancer

Colorectal cancer is the frequent cancer globally and has high mortality and incidence rates (Wieszczy et al., 2020; Świerczyński et al., 2021; Zaborowski et al., 2021; Zhao et al., 2021). Bi et al. (2020) revealed that LINC01116 was highly expressed in CRC tissues compared to normal tissues, and patients with high expression of LINC01116 had a very poor prognosis. LINC01116 knockdown substantially prevented the migration, proliferation, and invasion of CRC cells and activated cell apoptosis. Emerging evidence showed that a large number of patients were commonly diagnosed at terminal stages with high expression of LINC01116. Liang et al. (2021) identified that LINC01116 facilitated the growth of CRC cells and tumorigenicity through the downregulation of TPM1 expression. Specifically, LINC01116 can bind with EZH2 to accelerate the methylation of TPM1, which blocks the transcription of TPM1. Additionally, low expression of LINC01116 considerably impeded the tumorigenicity and angiogenesis of CRC cells in nude mice. Despite LINC01116 could serve as a diagnostic biomarker for in CRC, the deficiency of clinical application needs to be further investigated. For instance, researches of LINC01116 participated in CRC are only tested in tissues, and diverse effects between LINC01116 and molecular target markers are supposed to be probed in blood and other body fluids.

### Glioma

Brain gliomas are the most common primary malignant tumors in the central nervous system, presenting with an increasing mortality rate. Several patients present with an OS time of less than 2 years (Mondal and Kulshreshtha, 2021; Petridis et al., 2021; Tanabe et al., 2021; Tu et al., 2021). Wang et al. (2020c) found that the upregulation of LINC01116 in glioma cells is related to poor prognosis. When LINC01116 is knocked, G1-G0 phase arrest in Ln229 and U87 cells might be induced. Therefore, the apoptosis of glioma could be significantly inhibited. Moreover, LINC01116 has been shown to stimulate IL-1β transcription to generate an army of cytokines, which are associated with tumorigenicity *via* DDX5. Wang et al. (2020c) showed that LINC01116 positively regulates MDM2 to repress the p53 pathway, which activates the development of glioma cells. Current research shows that LINC01116 can substantially promote the proliferation and invasion abilities of glioma cells (Cole et al., 1986). Ye et al. revealed that LINC01116 expression is higher in glioma tissues than in normal tissues, and it could elevate the capacity of the cell cycle and cell proliferation by regulating the expression of VEGFA. In summary, these findings further clarify the biological functions of LINC01116 in glioma tumorigenesis and provide a promising treatment target for glioma patients.

### Osteosarcoma

Osteosarcoma is a leading cause of cancer-related death among young adolescents. Osteosarcoma patients retain high levels of metastasis and recurrence, accounting for approximately 30%–40% of cases (Lu et al., 2020; Zheng et al., 2020; Li et al., 2021c; Liu et al., 2021b). Zhang et al. (2019) reported that LINC01116 might remarkably accelerate the proliferation, migration, and invasion of osteosarcoma cells, but substantially precludes cell apoptosis. These results corroborated that LINC01116 directly interacted with EZH2 to mediate PTEN and p53. Thus, EZH2 knockdown reverses the LINC01116 functional effect on osteosarcoma cells (Zhang et al., 2019). Zhang et al. (2018) silenced LINC01116, inhibiting the viability of osteosarcoma cells and promoting cell apoptosis. Moreover, high expression of LINC01116 in patients generally results in a poorer prognosis. These results revealed a crucial role for LINC01116 in osteosarcoma. However, further investigation is required before clinical application.

### Other Cancers

Wu et al. (2020) shed new light on the silencing of LINC01116, which was shown to inhibit the tumorigenicity of OSCC through the upregulation of miRNA-136. Likewise, LINC01116 knockdown was shown to decrease the migration and invasion of HNSC cells, probably *via* the epithelial-mesenchymal transition pathway (Xing et al., 2020). Yu et al. (2021) suggested that LINC01116 was an oncogene that accelerates the development of prostate adenocarcinoma (PRAD) cells. Fang et al. (2018) reported that the proliferation and migration of EOC cells was increased with the overexpression of LINC01116. Knockdown of LINC01116 could function as an essential suppressor to block the viability and cloning ability of BC cells (Hu et al., 2018). Consequently, LINC01116 is a promising prognostic and therapeutic target for OSCC, HNSC, PRAD, EOC, and BC.

## Expression and Prognostic Value of LINC01116 in Cancers

**FIGURE 1:**
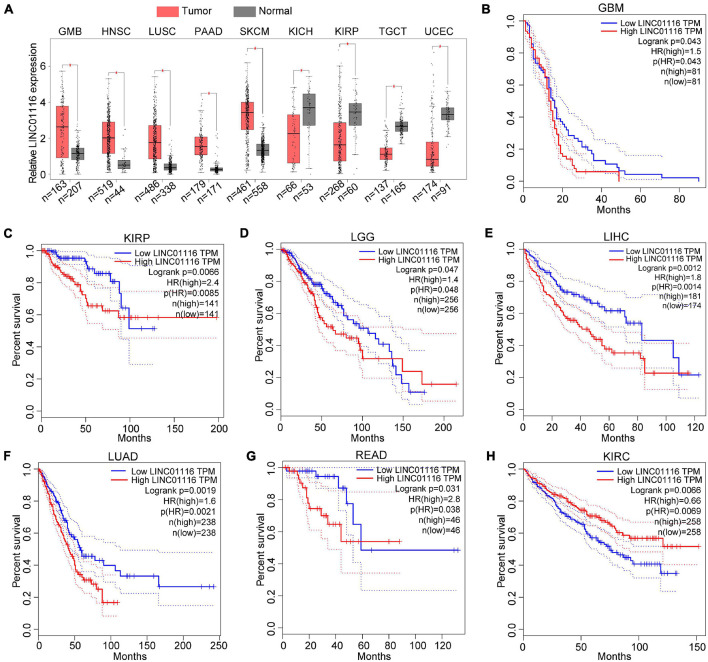
Expression and prognostic roles of LINC01116 in different cancer types. **(A)** Dysregulated expression of LINC01116 in GMB, HNSC, LUSC, PAAD, SKCM, KICH, KIRP, TGCT, and UCEC. **(B–G)** Patients with highly expressed LINC01116 had poor overall survival (OS) rate compared those with lowly expressed LINC01116 in GBM, KIRP, LGG, LIHC, LUAD, and READ. **(H)** Patients with decreased LINC01116 expression had poor overall survival (OS) rate compared those with increased LINC01116 expressed in KIRC.

To further assess the expression pattern of LINC01116 across pan-cancer, we used the GEPIA^1^ website to explore the expression level based on The Cancer Genome Atlas database (Tang et al., 2019). The results demonstrated that LINC01116 expression is upregulated in glioblastoma multiforme (GBM), head and neck squamous cell carcinoma (HNSC), lung squamous cell carcinoma (LUSC), pancreatic adenocarcinoma (PAAD), and skin cutaneous melanoma (SKCM). However, decreased expression of LINC01116 has been observed in kidney chromophobe (KICH), kidney renal papillary cell carcinoma (KIRP), testicular germ cell tumor (TGCT), and uterine corpus endometrial carcinoma (UCEC) (Figure 1A).

Likewise, we evaluated the effect of LINC01116 expression on the survival time of cancer patients. As shown in Figure 1B, when LINC01116 is highly expressed, patients with GBM generally have a shorter OS time than when the expression of LINC01116 is low. Similar results have been observed in KIRP, lower grade glioma (LGG), liver hepatocellular carcinoma (LIHC), lung adenocarcinoma (LUAD), and rectum adenocarcinoma (READ) (Figures 1C–G). Conversely, patients with decreased LINC01116 expression had a lower OS rate in kidney renal clear cell carcinoma (KIRC) (Figure 1H). Collectively, LINC01116 could be a novel biomarker for the diagnosis and prognostic determination of different cancer types.

## LINC01116 Influences Diverse Biological Functions in Cancers

**FIGURE 2:**
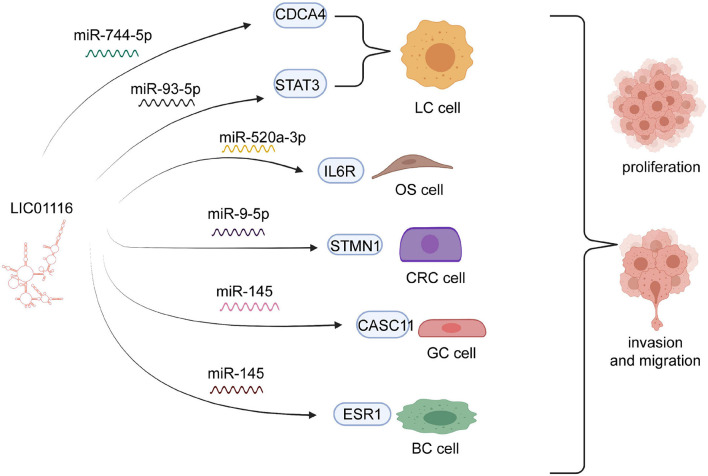
Diagram of the biological mechanisms involved in LINC01116 association with tumors. LINC01116 exerts an effect on the cell proliferation, invasion, and migration of multiple cancers, through sponging of miRNAs to mediate target regulators, such as miR-744-5p, miR-93-5p, miR-520a-3p, miR-9-5p, and miR-145.

LINC01116 has effects on multiple functions of tumors *via* complicated molecular mechanisms. To further explore these underlying processes, we summarize the complex molecular mechanisms in Table 2 and elucidate the association between LINC01116 and the biological functions of various cancers (Figure 2).

**TABLE 2:** The roles and functions of LINC01116 in cancers.

**Cancer type**	**Role**	**Related genes**	**Functions**	**PMID**
Lung cancer	Oncogene	miR-744-5P/CDCA4	Proliferation	34090440
		miR-93-5P/STAT3	Proliferation	33535997
		IFI44	Chemoresistance	31841994
Gastric cancer	Oncogene	miR-145/CASC11	Invasion and migration	31632064
Glioma	Oncogene	IL-1β	Proliferation and migration	32358484
		VEGFA	Proliferation, invasion, and migration	33760190
Osteosarcoma	Oncogene	miR-744-5P/MDM2	Proliferation	33760190
		miR-520a-3P/IL6R	Cell viability and migration	30098545
Colorectal cancer	Oncogene	TPM1	Proliferation and angiogenesis	33499872
		miR-9-5P/STMN1	Proliferation, invasion and migration	33116633
Oral squamous cell carcinoma	Oncogene	miRNA-136	Tumorigenicity	31452270
Neck squamous cell carcinoma	Oncogene	MYC	Migration and invasion	31703161
Prostate adenocarcinoma cell	Oncogene	/	Proliferation	33311496
Epithelial ovarian cancer	Oncogene	/	Proliferation and migration	30178832
Breast cancer	Oncogene	miR-145/ESR1	Proliferation	29687853

### Cell Proliferation, Invasion, and Migration

**FIGURE 3:**
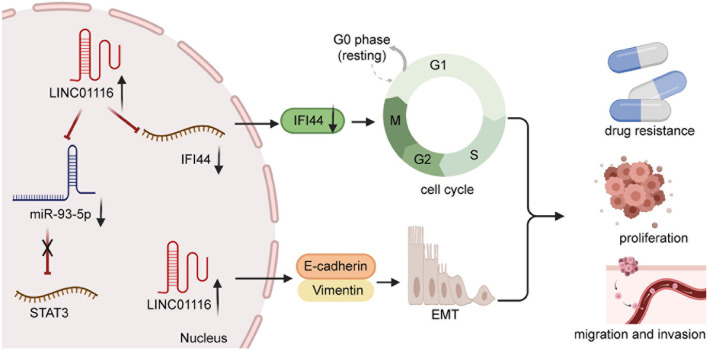
The overexpression of LINC00261 in non-small cell lung cancer accelerates tumor progression and chemoresistance.

**FIGURE 4:**
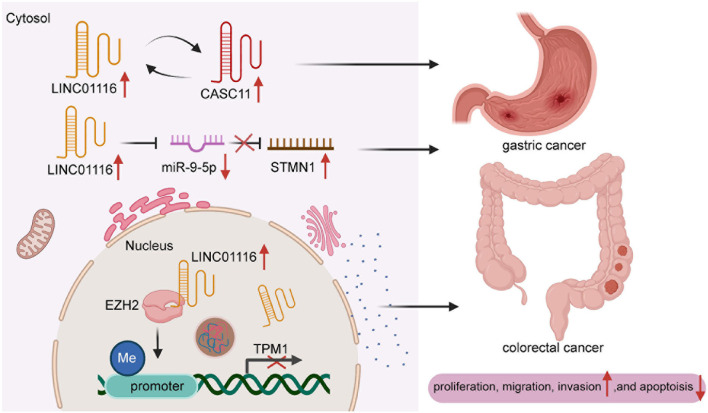
The molecular mechanism map of LINC00261 in gastric cancer and colorectal cancer.

Proliferation of cancer cells generally occurs rapidly, which markedly affects the prognosis of patients (Liu et al., 2019; Zhang et al., 2020). A recent study validated that miR-744-5p might be a novel target of LINC01116 in lung (LAD). miR-744-5p was known as the suppressor to involving in malignant tumors and negatively through regulating CDCA4. Knockdown of miR-744-5p could promote tumor cell proliferation and migration in LAD. LINC01116 could mediate the expression of CDCA4 by competitively binding the sites of CDCA4 with miR-744-5p, which markedly increased cell growth in LAD (Ren et al., 2021)Numerous experiments have shown that LINC01116 is overexpressed in small cell lung carcinoma (SCLC) and that it could upregulate STAT3 to boost SCLC cell invasion and migration. However, high expression of miR-93-5p suppresses the effect of LINC01116 overexpression. Thus, LINC01116 is likely to modulate miR-93-5p, which contributes to the expression levels of STAT3 (Figure 3). However, high expression of miR-93-5p and LINC01116 did not exert an influence on mutual expression, implying that miR-93-5p might have other targets (Xiang et al., 2017). In osteosarcoma cells, LINC01116 was shown to accelerate cell proliferation by targeting miR-520a-3p and upregulating IL6R (Zhang et al., 2018). Similarly, Liu et al. showed that LINC01116 interacts with CASC11, which regulates the invasion and migration of GC cells (Figure 4). Accumulating evidence suggests that miR-145 might bridge the interaction between LINC01116 and CASC11 (Su et al., 2019).

In nasopharyngeal carcinoma (NPC) cells, LINC01116 was shown to induce the translation of MYC and enhance the expression of MYC protein, which plays an essential role in proliferation. Likewise, when MYC remains highly expressed, the effect of LINC01116 deletion can be recovered to some degree (Tran et al., 2016). In CRC cells, LINC01116 negatively correlates with miR-9-5p regulation, promoting the proliferation, invasion, and migration of cancer cells. In contrast, miR-9-5p rescues the function of LINC01116. miR-9-5p tends to bind STMN1 to preclude its expression. LINC01116 partly regulates STMN1 to target miR-9-5p (Bi et al., 2020; Figure 4). Liang et al. reported that LINC01116 enhanced the CRC cell proliferation, invasion and migration through interacting with EZH2 to potentiate methylation in the TPM1 promoter region to suppress the transcription of TPM1 (Figure 4). In addition, a previous study confirmed that LINC01116 was a regulator of ESR1 related to the proliferation of BC cells by sponging miR-145. In brief, LINC01116 actively stimulates the development of cell proliferation in many cancers by prompting miRNAs to mediate the expression of several proteins.

### Chemoresistance

Chemotherapy is one of the primary treatment methods for several malignant tumors. However, the phenomenon of chemoresistance has increased, which has led to a serious dilemma in clinical treatment (Fatma et al., 2020; Jiang et al., 2020). Thus, there has been a great deal of research focusing on the molecular mechanisms of chemoresistance. Previous evidence has indicated that LINC01116 contributes to chemoresistance in some cancers (Li et al., 2021b), namely gefitinib and cisplatin resistance. Wang et al. (2020a) corroborated that LINC01116 contributed positively to the development of cisplatin resistance in LUAD, which depends on the EMT process (Figure 3). In contrast, LINC01116 silencing increases cisplatin sensitivity by mediating apoptosis and cell cycle distribution. Recent experiments have shed new light on the LINC01116-increasing effect of gefitinib resistance by regulating cell cycle through mediating IFI44 expression (Wang et al., 2020a; Figure 3). Furthermore, when LINC01116 is upregulated, the sensitivity of A549 cells to cisplatin is low. Conversely, silencing of LINC01116 generally leads to the inhibition of cisplatin resistance in A549 cells, *via* the stimulation of apoptosis and cell cycle arrest at the G0/G1 phase (Wang et al., 2020b). In summary, an abundance of chemoresistance mechanisms remain unclear. Thus, further studies are necessary.

## Conclusion

The improvement of research technology generates numerous possibilities for the study of lncRNAs. Substantial research has shown that lncRNAs are relevant to the biological process of tumor advancement as essential regulators of gene expression (Ramnarine et al., 2019; Olivero et al., 2020; Katsushima et al., 2021), and the dysregulation of LINC01116 has been identified as the promoter of the occurrence and progression of a variety of tumors. Plentiful expression of LINC01116 can be found in various cancers, such as lung cancer (Liu et al., 2021a; Mu et al., 2021; Zeng et al., 2021), gastric cancer, colorectal cancer, glioma, and osteosarcoma. When LINC01116 is highly expressed in multiple cancers, the survival time of these patients tends to be shorter. In several experiments, LINC01116 was found to be an independent prognostic factor in malignant tumors. Furthermore, accumulating evidence has revealed that LINC01116 can be regarded as a ceRNA that mediates gene expression by sponging miRNA, which plays an indispensable role in the proliferation, invasion, metastasis, chemoresistance, and apoptosis of tumors. For instance, LINC01116 functions as a regulator to positively promote the expression of STMN1 by interacting with miR-9-5p. In conclusion, we demonstrated that LINC01116 expression is linked to cancer, and LINC01116 has the potential of being a promising target in clinical tumor treatments. However, there are several dilemmas of LINC0116 applying to clinical treatment still need to be solved. Firstly, the molecular structure and functional information on LINC0116 remain uncharted. Without detailed understanding on the structure and functions of LINC0116, boosting LINC0116 -based therapy exists difficulty. Additionally, lncRNAs are considered to be weakly conserved across different species, the conversion from animal models to human clinical application might emerge block. Thus, it is necessary to further research to explore numerous mechanisms of LINC01116 associated with tumor biological processes.

## Author Contributions

YX, MZ, and XY drafted the manuscript. QZ and ZS drew the mechanism diagrams. YH and WG conceived of the study and guided the analysis. YH and YX edited and reviewed the manuscript. All authors read and approved the final manuscript.

## Conflict of Interest

The authors declare that the research was conducted in the absence of any commercial or financial relationships that could be construed as a potential conflict of interest.

## Publisher’s Note

All claims expressed in this article are solely those of the authors and do not necessarily represent those of their affiliated organizations, or those of the publisher, the editors and the reviewers. Any product that may be evaluated in this article, or claim that may be made by its manufacturer, is not guaranteed or endorsed by the publisher.

## Footnotes

1
http://gepia.cancer-pku.cn/
